# Global Research Trends in Immunotherapies for Recurrent Pregnancy Loss: A Bibliometric Analysis

**DOI:** 10.5935/1518-0557.20250188

**Published:** 2026

**Authors:** Marcelo Borges Cavalcante, Daniel de Sousa Sobral, Raíssa Helen de Andrade Praciano, Maria Edith Holanda Banhos, Cristiana Libardi Miranda Furtado

**Affiliations:** 1 Graduate Program in Medical Sciences, Universidade de Fortaleza (UNIFOR), Fortaleza, CE, Brazil; 2 Medical School, Universidade de Fortaleza (UNIFOR), Fortaleza, CE, Brazil; 3 CONCEPTUS - Reproductive Medicine, Fortaleza, CE, Brazil; 4 Department of Genetics, Ecology and Evolution, Institute of Biological Sciences, Federal University of Minas Gerais, Belo Horizonte, MG, Brazil

**Keywords:** bibliometric analysis, habitual abortion, recurrent miscarriage, immunotherapy, recurrent pregnancy loss

## Abstract

This bibliometric study investigates global research trends in the application of immunotherapies for managing recurrent pregnancy loss (RPL). Data were retrieved from the Web of Science (WoS) database, encompassing 1,735 publications from 1964 to 2024. The analysis demonstrates a steady increase in research activity, with an average annual growth rate of 1.14%. Leading journals in the field include *The American Journal of Reproductive Immunology and Human Reproduction*, while prominent contributors such as Ole Bjarne Christiansen and William H. Kutteh have significantly influenced the domain. The United States, China, and the United Kingdom were identified as the most productive countries in terms of publication volume and citation metrics. Keyword co-occurrence analysis reveals a thematic progression from early-stage experimental treatments to a more advanced focus on immunological and molecular mechanisms underlying RPL. Among various therapeutic modalities, heparin, aspirin, and intravenous immunoglobulin have been the most frequently studied. Meanwhile, novel immunotherapies are emerging as promising alternatives, although their clinical efficacy remains to be thoroughly validated. This study emphasizes the critical role of international collaboration in advancing the field and highlights persistent research gaps that warrant further investigation. Overall, the findings provide a comprehensive overview of the evolution, current status, and future directions of immunotherapy research in the context of recurrent pregnancy loss..

## INTRODUCTION

Recurrent pregnancy loss (RPL), also referred to as recurrent miscarriage or habitual abortion, was traditionally defined as the occurrence of three or more consecutive spontaneous pregnancy losses during the first half of gestation (WHO, 1977). However, in light of evolving reproductive patterns - such as delayed maternal age at first pregnancy and rising obesity rates - the currently accepted definition includes two or more consecutive pregnancy losses (ESHRE Guideline Group on RPL *et al.*, 2023).

RPL affects approximately 2%-4% of couples of reproductive age, with recent studies indicating a rising incidence (Genovese & McQueen, 2023). Known etiological factors include parental chromosomal abnormalities, anatomical defects, endocrine disorders, environmental exposures, antiphospholipid syndrome (APS), and obesity, which together account for up to 50% of cases (ESHRE Guideline Group on RPL *et al.*, 2023). However, in the remaining cases, the underlying cause remains unidentified, underscoring RPL as a persistent challenge in reproductive medicine (ESHRE Guideline Group on RPL *et al.*, 2023).

The maternal immune response during embryo implantation plays a pivotal role in pregnancy success and has been extensively studied since Peter Medawar’s seminal work. Emerging evidence suggests that a failure in maternal immune tolerance toward the embryonic semi-allograft contributes to reproductive failures, including RPL and recurrent implantation failure (Billington, 2003; Petroff *et al.*, 2022). Specifically, dysregulation of immune mechanisms - such as heightened T-helper 1 (Th1) and Th17 responses, reduced regulatory T cell (Treg) activity, increased natural killer (NK) cell cytotoxicity, aberrant macrophage function, and diminished HLA-G expression - has been associated with inflammatory responses that compromise maternal-fetal tolerance and lead to pregnancy loss (Kwak-Kim *et al.*, 2022; Cavalcante *et al.*, 2025).

Immunotherapies have thus been proposed for RPL cases associated with autoimmune disorders, immune dysfunction, or idiopathic causes. Advances in the understanding of immune-mediated reproductive failures, coupled with improvements in immunodiagnostic tools, have facilitated the development of personalized immunological treatment strategies, many of which have shown promising clinical outcomes (Kwak-Kim *et al.*, 2022; Cavalcante *et al.*, 2023a).

Bibliometric analyses in reproductive medicine are valuable for tracking research trends, evaluating scientific output, and identifying knowledge gaps (Aleixandre-Benavent *et al.*, 2015; Meng *et al.*, 2022; Montazeri *et al.*, 2023). Such analyses help determine which topics receive the most attention, which methodologies dominate the field, and where further investigation is needed (Montazeri *et al.*, 2023). Moreover, bibliometric insights can inform public policy, guide clinical practice, and enhance the dissemination of evidence-based interventions (Montazeri *et al.*, 2023). Mapping collaboration networks among researchers and institutions also supports the development of multidisciplinary approaches essential for addressing complex reproductive health challenges.

The present bibliometric analysis investigates the global scientific landscape of immunotherapies for RPL. It aims to identify leading research trends, key contributors, productive countries, and influential journals. Additionally, this study explores frequently used keywords to gain insight into the immunological mechanisms underlying RPL and the immunotherapeutic strategies most commonly employed in its management.

## METHODS

This bibliometric analysis was conducted in accordance with the Guideline for Reporting Bibliometric Reviews of Biomedical Literature (BIBLIO) (Montazeri *et al.*, 2023). As the study exclusively utilized data from previously published and indexed scientific literature, it did not involve human participants, primary data collection, or sensitive information and thus did not require ethical approval.

### Data sources and search strategy

The literature search was performed using the Web of Science (WoS) database, which is widely regarded as the gold standard for bibliometric analyses due to its rigorous journal selection criteria, long-standing indexing history (since 1964), and compatibility with analytical tools. WoS is broadly recognized by academic institutions, research funding bodies, and global ranking systems for its quality-controlled and standardized datasets. Furthermore, its data export functions facilitate integration with bibliometric software such as VOSviewer and Bibliometrix, making it ideal for comprehensive trend and network analyses.

The search strategy employed the following terms: (“immunotherapy” OR “immune therapy” OR “immunotherapies” OR “corticosteroids” OR “corticosteroid” OR “prednisone” OR “prednisolone” OR “intravenous immunoglobulin” OR “immunoglobulin” OR “IVIG” OR “lymphocyte immunotherapy” OR “allogeneic lymphocytes immunotherapy” OR “intravenous lipids emulsion” OR “lipids emulsion” OR “intralipid” OR “vitamin D” OR “calcineurin inhibitors” OR “cyclosporine” OR “tacrolimus” OR “granulocyte-colony stimulating factor” OR “G-CSF” OR “granulocyte-macrophage colony stimulating factor” OR “GM-CSF” OR “tumor necrosis factor antagonists” OR “tumor necrosis factor” OR “TNF-α” OR “heparin” OR “hydroxychloroquine” OR “chloroquine” OR “human chorionic gonadotropin” OR “hCG”) AND (“recurrent miscarriage” OR “recurrent abortion” OR “habitual abortion” OR “recurrent pregnancy loss”).

**Figure 1:**
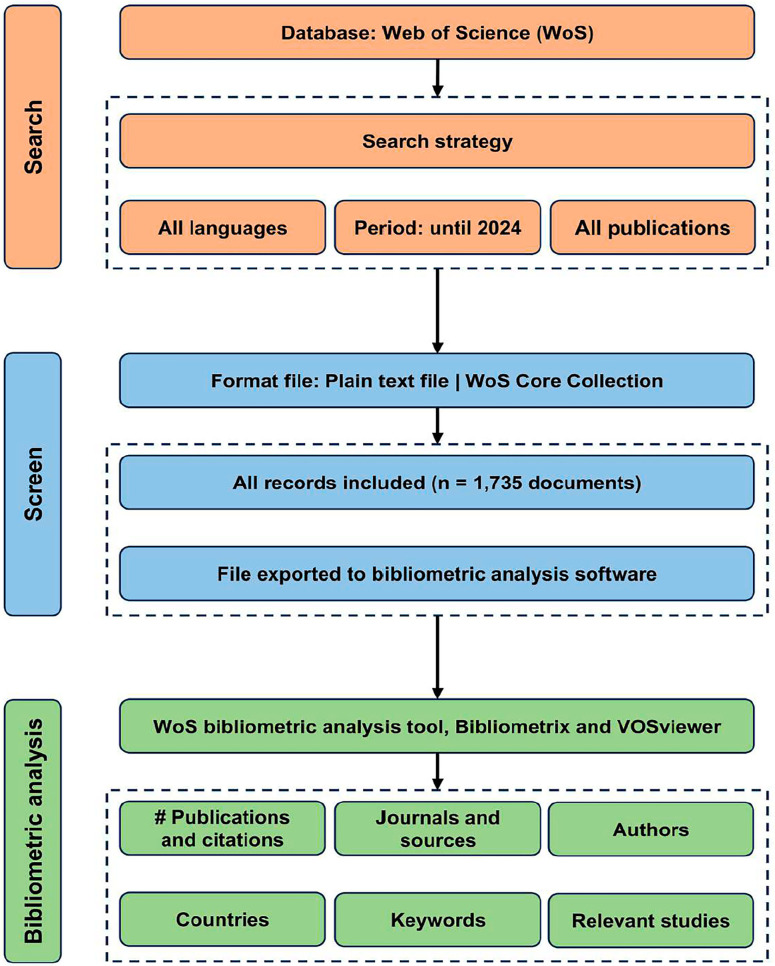
Flowchart of the bibliometric analysis of immunotherapies in recurrent pregnancy loss (RPL) management. This flowchart illustrates the methodology used to perform a bibliometric analysis of immunotherapy applications in managing recurrent pregnancy loss. The process commenced with a comprehensive search of the Web of Science (WoS) database, encompassing all languages and publication years up to 2024. A total of 1,735 documents were identified and exported for analysis using Bibliometrix and VOSviewer software. The analysis assessed publication and citation metrics, prominent journals, authors, contributing countries, and keyword trends to map the development and current landscape of research in this field.

No language restrictions were applied. All document types available up to December 31, 2024, were included. The retrieved records were exported in .TXT format for further processing using VOSviewer and the Bibliometrix R package (Figure 1).

### Data analysis and tools

The dataset was analyzed using the built-in tools of WoS, the Bibliometrix R package, and VOSviewer. Bibliometrix facilitated the generation of descriptive statistics, productivity indices, and citation metrics, while VOSviewer was employed to construct network visualizations.

Journal and publication metrics were evaluated based on the total number of articles published per journal, local citation counts (i.e., citations within the dataset), and Journal Impact Factors (JIFs) derived from the 2023 WoS database, including quartile rankings. The publication year of the first indexed article (PY Start) was also noted to identify the onset of research activity in each source.

Author productivity was assessed using the total number of publications per author in WoS, local citation counts, PY Start, and three key impact metrics: h-index (the largest *h* such that *h* publications have at least *h* citations each), g-index (the highest number *g* such that the top *g* publications received at least *g^2^* citations combined), and m-index (the h-index normalized by career duration, calculated as *h*/*n*, where *n* is the number of years since the author’s first publication).

To evaluate international research collaboration, the Country Scientific Production (CSP) metric was employed to quantify the total number of publications per country, accounting for all types of authorship (i.e., first author, co-author, and corresponding author). The corresponding author’s country was used as a proxy to determine global leadership in the field. Research output was further classified into Single Country Publications (SCP) - representing studies authored solely by researchers from one country, indicating national scientific productivity without international collaboration - and Multiple Country Publications (MCP) - comprising publications co-authored by researchers from institutions in two or more countries, reflecting international collaboration. A high number of MCPs indicates strong integration with the global scientific community.

To identify the most influential studies on immunotherapies in the management of RPL, both local and global citation counts were considered. These top-cited studies were then ranked and listed alongside additional variables such as author, publication year, title, DOI, and journal. To assess thematic structures and research trends, VOSviewer was utilized for two key bibliometric analyses. The keyword co-occurrence network revealed the most frequently used keywords in RPL immunotherapy research, grouping them into color-coded clusters based on their co-occurrence in publications. This visualization illuminated the dominant research themes and their interconnections. The temporal evolution analysis used a blue-to-yellow color gradient to show how research foci have shifted over time, from early experimental immunotherapies in the 1990s-2000s to more recent investigations into molecular mechanisms, genetic factors, and metabolic influences in the 2010s-2020s.

## RESULTS

### Characteristics of scientific production

**Figure 2:**
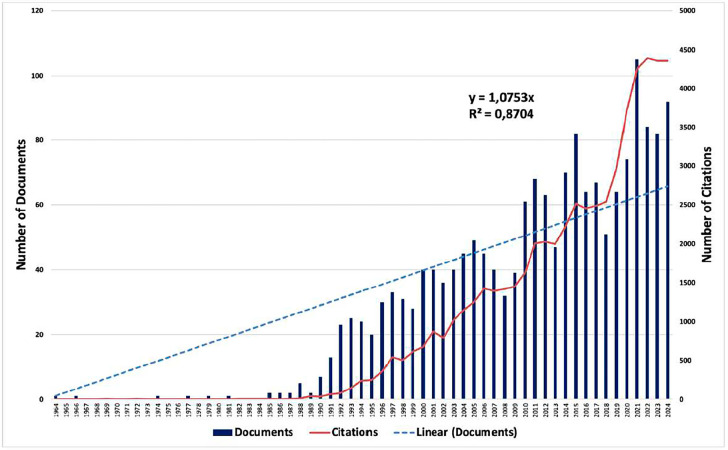
Annual trends in publications and citations on immunotherapies for RPL. This figure presents the annual growth in the number of publications (blue bars) and citations (red line) related to immunotherapy research in RPL. The trend line (*y* = 1.0753x, *R^2^* = 0.8704) indicates a steady increase in scientific output and impact, underscoring the growing academic interest in this topic over time.

A bibliometric analysis of literature retrieved from WoS identified a total of 1,735 documents on immunotherapies for RPL, published between 1964 and 2024 across 442 sources. A notable upward trend in publication volume and citations was observed. The publication trend follows the equation *y*=1.0753*x*, with a coefficient of determination (*R*^2^=0.8704), indicating a strong positive correlation and consistent growth in research output (Figure 2). The annual growth rate in scientific production was 1.14%, with visible surges particularly after the years 1990 and 2010.

Of the total publications, original articles accounted for 66.7%, literature reviews for 20.4%, and other publication types - such as meeting abstracts, proceedings papers, editorials, letters, retracted publications, notes, and expressions of concern - for 12.9%. The total citation count reached 58,273, averaging 33.88 citations per document, based on an analysis of 38,376 references. The average age of documents was 13.9 yr, indicating a mix of foundational and recent studies (see Supporting Information Data S1).

Most documents were published in English (97.9%), followed by German (1.3%), French (0.4%), Spanish (0.3%), and Polish (0.1%). The WoS categorized these documents into several subject areas: Obstetrics and Gynecology (25.1%), Reproductive Biology (23.5%), Immunology (14.9%), Hematology (5.3%), General Internal Medicine (5.3%), Peripheral Vascular Disease (3.4%), and Others (22.5%; see Data S1).

### Journals and scientific sources

Table 1 presents the leading journals publishing research on immunotherapies for RPL, including bibliometric indicators such as the number of articles published, local citation count, JIF, JIF ranking, quartile classification, scientific productivity indices (h-index, g-index, and m-index), and the year of the journal’s first relevant publication in this area.

**Table 1:** Leading journals publishing research on immunotherapies for recurrent pregnancy loss.

Sources	No. documents	Local citation	JIF	JIF rank	JIF Quartile	h-index	g-index	m-index	PY start
Am J Reprod Immunol	166	5256	2.5	20/39^ª^	Q3	42	61	1.166	1990
Hum Reprod	105	5062	6.0	4/39^ª^	Q1	42	68	1.135	1989
J Reprod Immunol	105	1855	2.9	14/39^ª^	Q2	26	41	0.742	1991
Fertil Steril	93	4259	6.6	3/39^ª^	Q1	33	66	0.970	1992
Obstet Gynecol	29	1872	5.5	7/136^b^	Q1	21	29	0.466	1981
Am J Obstet Gynecol	26	3513	8.7	2/136^b^	Q1	18	26	0.290	1964
Thromb Res	23	547	3.7	23/97^c^	Q1	9	17	0.321	1998
Eur J Obstet Gynecol Reprod Biol	22	694	2.1	58/136^b^	Q2	11	21	0.268	1985
Lupus	21	962	1.9	28/57^d^	Q2	13	21	0.419	1995
J Obstet Gynaecol Res	20	208	1.6	82/136^b^	Q3	10	20	0.454	2004

No. documents: total number of documents published by the journal in documents indexed in WoS between 1964 and 2024. Local citations: citations from within the search result in the WoS database, citations occurred in a total of 1735 publications. JIF: The Journal Impact factor. JIF, JIF rank and quatile metrics were extracted from the 2023 results. PY start: publication year start (year of the first publication on this topic in the journal). Am J Reprod Immunol: American Journal of Reproductive Immunology; Hum Reprod: Human Reproduction; J Reprod Immunol: Journal of Reproductive Immunology; Fertil Steril: Fertility and Sterility; Obstet Gynecol: Obstetrics and Gynecology; Am J Obstet Gynecol: American Journal of Obstetrics and Gynecology; Thromb Res: Thrombosis Research; Eur J Obstet Gynecol Reprod Biol: European Journal of Obstetrics & Gynecology and Reproductive Biology; J Obstet Gynaecol Res: Journal of Obstetrics and Gynaecology Research. a: position of the journal in the reproductive biology area ranking. b: position of the journal in the Obstetrics & Gynecology area ranking. c: position of the journal in the hematology area ranking. d: position of the journal in the rheumatology area ranking.

The *American Journal of Reproductive Immunology* (*AJRI*), the official journal of the American Society for Reproductive Immunology, ranks first with 166 publications and 5,256 local citations, establishing itself as a key journal in the field. Despite its modest JIF of 2.5 (ranked 20th out of 39 in reproductive biology), *AJRI* is recognized for its focused expertise at the intersection of immunology and reproduction. Its h-index of 42 and g-index of 61 reflect substantial scientific relevance.

*Human Reproduction*, the journal of the European Society for Human Reproduction and Embryology, and the *Journal of Reproductive Immunology*, published by the European Society for Reproductive Immunology, are tied for second place with 105 articles each. However, *Human Reproduction* exhibits a significantly greater impact, with 5,062 local citations and a JIF of 6.0, ranking fourth in reproductive biology. In contrast, the *Journal of Reproductive Immunology* holds a JIF of 2.9, ranks 14th in the same category, and is classified in Q2, indicating a moderate but still relevant impact.

*Fertility and Sterility*, published by the American Society for Reproductive Medicine, also stands out with 93 articles and 4,259 local citations. With a JIF of 6.6, it ranks third in reproductive biology and is classified in Q1, underscoring its high visibility and influence, particularly in assisted reproduction and fertility immunology.

*Obstetrics and Gynecology* and the *American Journal of Obstetrics and Gynecology* have fewer publications (29 and 26, respectively) but demonstrate high impact through their JIFs of 5.5 and 8.7. The latter ranks second out of 136 in gynecology and obstetrics and holds the highest impact factor among all journals analyzed, serving as a leading reference for clinical research in the field.

Other journals, including *Thrombosis Research*, the *European Journal of Obstetrics & Gynecology and Reproductive Biology, Lupus*, and the *Journal of Obstetrics and Gynecology Research*, contributed a smaller number of publications and citations. Nevertheless, they play a valuable role in advancing knowledge on immunotherapies for RPL, particularly within niche areas such as thrombophilia, autoimmune disorders, and vascular complications associated with pregnancy loss.

### Most influential authors

Table 2 presents the top 10 most productive authors in the field of immunotherapies for RPL, based on key bibliometric indicators, including number of publications, h-index, g-index, m-index, and local citation count. These metrics provide insight into each author’s scientific output and the impact of their work.

**Table 2:** Most productive authors in immunotherapies for RPL.

Authors	No. documents	Local citation	h-index	g-index	m-index	PY start
Christiansen OB	47	398	21	35	0.567	1989
Kutteh WH	34	827	24	33	0.727	1993
Branch DW	33	487	20	31	0.526	1988
Regan L	32	891	24	32	0.685	1991
Quenby S	32	477	22	30	0.687	1994
Middeldorp S	28	424	18	28	0.818	2004
Kwak-Kim J	22	255	15	20	0.789	2007
Yamada H	22	94	9	19	0.360	2001
Clark DA	20	239	14	20	0.437	1994
Goddijn M	20	381	15	20	0.882	2009

No. documents: total number of documents published by the author in journals indexed in WoS between 1964 and 2024. Local citations: citations from within the search result in the WoS database, citations occurred in a total of 1735 publications. PY start: publication year start (year of the first publication on this topic by the author).

Ole Bjarne Christiansen ranks as the most prolific author, with 47 publications since 1989. His h-index of 21 indicates that 21 of his articles have each received at least 21 citations. A g-index of 35 reflects the weight of his most cited works, while an m-index of 0.567 points to a consistent scholarly impact over time. Although his local citation count (398) is lower than some peers, his sustained productivity marks a substantial contribution to the field.

William H. Kutteh and Lesley Regan are distinguished by the highest h-index (24) among the group, indicating broad recognition of their research. Lesley Regan leads in total local citations (891), underscoring her prominent influence, while William H. Kutteh follows closely with 827 citations. Both began publishing in the early 1990s and have m-index values above 0.68, demonstrating sustained and influential academic trajectories.

D. Ware Branch has contributed 33 publications since 1988, with an h-index of 20, g-index of 31, and an m-index of 0.526, suggesting moderate but steady scholarly impact over time. Among researchers who started publishing post-1990, Siobhan Quenby stands out with 32 articles, an h-index of 22, and a g-index of 30, along with a high m-index of 0.687, indicating consistent and growing influence.

Saskia Middeldorp, who began publishing in 2004, has an m-index of 0.818, the highest among the top authors except one, suggesting a rapid rise in impact within a relatively short period. Joanne Kwak-Kim, Hideto Yamada, David A. Clark, and Mariette Goddijn round out the top contributors. Although they have slightly lower hand g-indices, their work remains influential. Notably, Mariette Goddijn, who started publishing in 2009, has an m-index of 0.882, the highest among all authors listed, reflecting a strong and accelerating scientific trajectory.

### Countries, coauthorship, and international collaboration analysis

Table 3 provides a comprehensive analysis of the countries contributing most significantly to research on immunotherapies for RPL. Key bibliometric indicators include total scientific output (CSP), local citation count, average citations per article, share of corresponding author contributions, and the classification of articles as SCPs or MCPs, reflecting international collaboration.

**Table 3:** Countries with the highest research output on immunotherapies for RPL.

Country	CSP	Local citations	Average Article Citation	CAC (%)	SCP	MCP	MCP/SCP
USA	630	13454	48.9	275 (15.8)	219	56	0.25
China	532	2242	11.4	197 (11.3)	171	26	0.15
United Kingdom	480	9803	64.9	151 (8.7)	119	32	0.26
Japan	329	2471	22.5	110 (6.3)	104	6	0.05
Italy	298	1990	28.8	69 (3.8)	54	15	0.27
Canada	267	2906	53.8	54 (3.1)	40	14	0.35
Spain	250	1954	43.4	45 (2.6)	37	8	0.21
France	236	1496	28.8	52 (2.9)	41	11	0.26
Germany	234	1699	21.8	78 (4.5)	55	23	0.41
Iran	217	766	19.6	39 (2.2)	33	6	0.18

CSP: Country Scientific Production, total number of publications by country, considering any authorship (first author, co-author or corresponding author). CAC: corresponding author's countries: Identifies the country of affiliation of the corresponding author (the one who leads communication with the journal). SCP: single country publication; MCP: multiple country publication.

The United States leads in scientific output, with 630 publications, the highest local citation count (13,454), and an average of 48.9 citations per article. It also accounts for the largest number of articles with corresponding authorship (275 publications, 15.8% of the total). Although the United States engages in international collaboration (56 MCPs), its MCP/SCP ratio of 0.25 suggests a moderate level of global research cooperation.

The People’s Republic of China ranks second in output, with 532 publications. However, it exhibits a lower scholarly impact, as evidenced by 2,242 local citations and an average of 11.4 citations per article. Furthermore, its MCP/SCP ratio of 0.15 reflects a limited degree of international collaboration, indicating that most research is conducted domestically.

In contrast, the United Kingdom is notable for producing high impact research, with an average of 64.9 citations per article, the highest among all countries analyzed. With 480 publications, the United Kingdom shows strong academic influence and collaboration, as demonstrated by its MCP/SCP ratio of 0.26.

Japan has contributed 329 publications, with a moderate impact (22.5 citations per article) but one of the lowest collaboration levels (MCP/SCP = 0.05), suggesting that its research is primarily nationally focused. Italy, with 298 publications and 28.8 citations per article, shows slightly more collaboration (MCP/SCP = 0.27) but still leans toward internal research activity.

Canada stands out for both impact and collaboration. With 267 publications and an average of 53.8 citations per article, it also boasts one of the highest MCP/SCP ratios (0.35), indicating significant international integration. Similarly, Spain and France, with 250 and 236 publications respectively, show moderate collaboration levels (MCP/SCP ratios of 0.21 and 0.26, respectively).

Germany is particularly distinguished by its MCP/SCP ratio of 0.41, the highest among all countries listed, highlighting its strong commitment to international cooperation in RPL research. However, Iran, with 217 publications, shows relatively low scholarly influence (19.6 citations per article) and a limited degree of collaboration (MCP/SCP = 0.18), suggesting a predominance of domestically conducted research.

### Key research topics and thematic evolution

The bibliometric analysis of all keywords (including both author and Plus keywords) identified 2,764 unique terms, revealing prevailing research trends in the study of immunotherapies for RPL. Among keywords associated with the etiology of RPL, “natural killer cells” emerged as the most frequently occurring term (185 occurrences), followed by “antiphospholipid antibodies” (107 occurrences) and “antiphospholipid antibody syndrome” (96 occurrences). Other frequently used terms included “cytokine” (86 occurrences), “tumor necrosis factor” (60 occurrences), “regulatory T-cells” (56 occurrences), “autoimmunity” (51 occurrences), and “T-cells” (42 occurrences), underscoring the central role of immune dysregulation in the pathogenesis of RPL.

Regarding therapeutic interventions, the most common keywords linked to immunotherapy use in RPL management were “heparin” (261 occurrences), “aspirin” (194 occurrences), and “intravenous immunoglobulin (IVIG)” (174 occurrences). Additional keywords included “corticosteroids” (44 occurrences), “lymphocyte immunotherapy (LIT)” (35 occurrences), and “hydroxychloroquine (HCQ)” (16 occurrences), reflecting a wide spectrum of therapeutic modalities investigated in this field.

**Figure 3:**
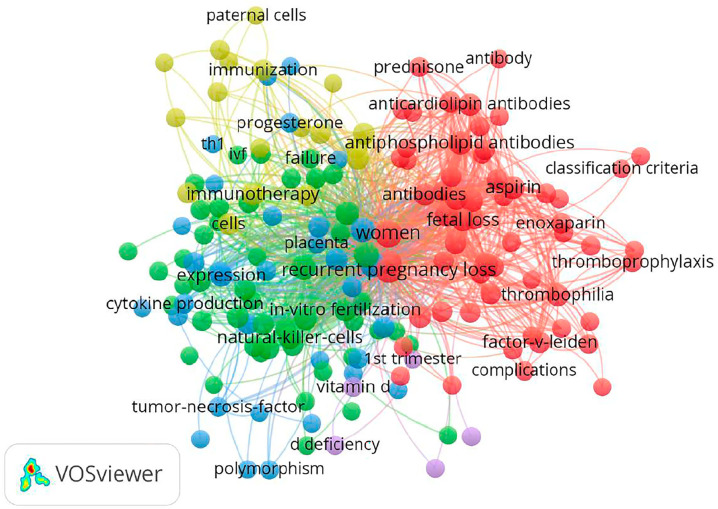
Keyword co-occurrence network in immunotherapy research for RPL. This VOSviewer-generated network visualizes the co-occurrence of keywords in the analyzed literature on immunotherapies for RPL. Keywords are grouped into thematic clusters based on their frequency and co-appearance in publications, with colors denoting distinct research themes: red cluster (focused on thrombophilia, antiphospholipid antibodies, anticoagulant therapies [e.g., aspirin and enoxaparin], and pregnancy complications); green cluster (includes terms related to immunotherapeutic strategies such as natural killer [NK] cells, cytokines, and *in vitro* fertilization [IVF]); yellow cluster (pertains to paternal cell immunization, progesterone, and IVF failure); blue cluster (covers immune response elements such as tumor necrosis factor, vitamin D, and immunogenetic polymorphisms); and purple cluster (represents emerging or less commonly studied concepts in immunogenetics and immunological susceptibility to pregnancy loss). Node size indicates keyword frequency, while edge thickness denotes the strength of co-occurrence. Central nodes represent broader, widely discussed topics, whereas peripheral nodes reflect niche areas of research.

The co-occurrence network of keywords, generated using VOSviewer, provides a visual representation of research themes and their interconnections. The network categorizes related terms into thematic clusters, each indicating a distinct focus within the broader research landscape (Figure 3). The red cluster groups terms related to thrombophilia, antiphospholipid antibodies, and anticoagulant therapies (e.g., aspirin and enoxaparin), emphasizing their relevance in the management of immune-mediated pregnancy complications. The green cluster centers on immunotherapeutic strategies, highlighting key concepts such as NK cells, cytokine profiles, and *in vitro* fertilization (IVF), illustrating the intersection between immunology and assisted reproductive technologies. The yellow cluster includes keywords linked to paternal cell immunization, progesterone supplementation, and IVF failure, suggesting their therapeutic relevance in certain subpopulations of RPL patients. The blue cluster reflects inflammatory and immunogenetic mechanisms, with keywords such as tumor necrosis factor (TNF), vitamin D, and polymorphisms, pointing to molecular and environmental contributors to pregnancy loss. The purple cluster includes emerging and less frequent keywords, potentially indicating novel directions in the study of genetic predispositions and immune pathways associated with RPL.

**Figure 4:**
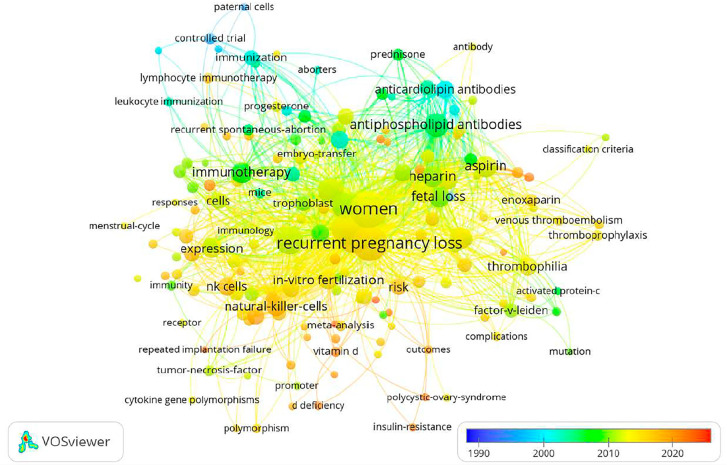
Temporal evolution of keywords in immunotherapy research for RPL. This figure illustrates the temporal dynamics of keyword emergence in immunotherapy-related RPL research using a VOSviewer-based overlay visualization. The color gradient (blue to yellow) reflects the chronological appearance of keywords, with blue representing earlier research focus (1990s-2000s) and yellow indicating recent trends (2010s-2020s). Early phase (1990s-2000s): Emphasis on paternal cell immunization, leukocyte and lymphocyte immunotherapy, controlled trials, and antiphospholipid/anticardiolipin antibodies as early experimental approaches to immune modulation in RPL. Intermediate phase (2000s-2010s): Focus shifted toward immune regulation, particularly cytokines, NK cells, and their relevance in IVF and embryo transfer. Coagulation-related terms such as Factor V Leiden, thromboprophylaxis, heparin, and aspirin also gained prominence. Recent phase (2010s-2020s): Research has increasingly examined genetic and metabolic contributions to RPL, including vitamin D, cytokine gene polymorphisms, insulin resistance, and polycystic ovary syndrome. The rising use of meta-analyses and systematic reviews reflects a transition to evidence-based evaluation of immunotherapies. Topics like repeated implantation failure, tumor necrosis factor, and immune receptor expression suggest deeper exploration of molecular mechanisms underlying RPL.

The temporal evolution of keywords further elucidates how research focus has shifted over the past three decades (Figure 4). In the early phase (1990s-2000s), studies largely concentrated on paternal cell and leukocyte immunization and lymphocyte immunotherapy, reflecting early experimental efforts to modulate maternal immune tolerance. During this period, antiphospholipid and anticardiolipin antibodies also gained attention for their diagnostic and therapeutic relevance.

In the intermediate phase (2000s-2010s), research emphasis expanded to include cytokines, NK cells, and the regulation of immune responses. Simultaneously, studies began incorporating assisted reproductive technologies, particularly IVF and embryo transfer, into the context of RPL. This period also saw increased exploration of thrombophilia and anticoagulant interventions, with frequent mention of Factor V Leiden, heparin, enoxaparin, and thromboprophylaxis as possible therapeutic measures.

In recent years (2010s-2020s), attention has turned to genetic and metabolic contributors to RPL, including vitamin D deficiency, cytokine gene polymorphisms, and insulin resistance, especially in patients with polycystic ovary syndrome. The growing presence of systematic reviews and meta-analyses reflects an increasingly evidence-based approach to assessing the efficacy of immunotherapies. Additionally, emerging terms such as repeated implantation failure, immune receptor expression, and tumor necrosis factor indicate a deeper investigation into the molecular and immunogenetic mechanisms underlying pregnancy loss.

### Most relevant publications

Table 4 presents a selection of the most frequently cited articles in the field of immunotherapies for RPL, highlighting the scientific impact of each study. Two key bibliometric indicators are used: local citations, which measure relevance within the specific context of immunotherapy for RPL, and global citations, which reflect broader influence across the international scientific community.

**Table 4:** Most productive authors in immunotherapies for RPL.

Author	Year	Title	DOI	Journal	Local Citation	Global Citation
Rai R *et al*.	1997	Randomized controlled trial of aspirin and aspirin plus heparin in pregnant women with recurrent miscarriage associated with phospholipid antibodies (or antiphospholipid antibodies)	10.1136/bmj.314.7076.253	BMJ	300	818
Kutteh WH	1996	Antiphospholipid antibody-associated recurrent pregnancy loss: treatment with heparin and low-dose aspirin is superior to low-dose aspirin alone	10.1016/s0002-9378(96)70610-5	Am J Obstet Gynecol	254	619
Kaandorp SP *et al*.	2010	Aspirin plus heparin or aspirin alone in women with recurrent miscarriage	10.1056/NEJMoa1000641	NEJM	153	328
Farquharson RG *et al*.	2002	Antiphospholipid syndrome in pregnancy: a randomized, controlled trial of treatment	10.1016/S0029-7844(02)02165-8	Obstet Gynecol	134	356
Clark P *et al*.	2010	SPIN (Scottish Pregnancy Intervention) study: a multicenter, randomized controlled trial of low-molecular-weight heparin and low-dose aspirin in women with recurrent miscarriage	10.1182/blood-2010-01-267252	Blood	99	186
Empson M *et al*.	2005	Prevention of recurrent miscarriage for women with antiphospholipid antibody or lupus anticoagulant	10.1002/14651858.CD002859.pub2	Cochrane Database Syst Rev	96	337
Girardi G *et al*.	2004	Heparin prevents antiphospholipid antibody-induced fetal loss by inhibiting complement activation	10.1038/nm1121	Nat Med	87	504
Rai RS *et al*.	1995	High prospective fetal loss rate in untreated pregnancies of women with recurrent miscarriage and antiphospholipid antibodies	10.1093/oxfordjournals.humrep.a135907	Hum Reprod	86	207
Brenner B *et al*.	2000	Gestational outcome in thrombophilic women with recurrent pregnancy loss treated by enoxaparin	10.1055/s-0037-1613894	Thromb Haemost	86	244
Branch DW *et al*.	1992	Outcome of treated pregnancies in women with antiphospholipid syndrome: an update of the Utah experience	NA	Obstet Gynecol	83	382

BMJ: British Medical Journal; Am J Obstet Gynecol; NEJM: The New England Journal of Medicine; Cochrane Database Syst Rev: The Cochrane database of systematic reviews Nat Med: Nature medicine; Hum Reprod: Human Reproduction; Thromb Haemost: Thrombosis and Haemostasis; Obstet Gynecol: Obstetrics and Gynecology. NA: Not available.

The most highly cited publication is the randomized clinical trial (RCT) by Rai *et al*. (1997), published in the *British Medical Journal*, which received 300 local citations and 818 global citations. This seminal study compared aspirin alone versus aspirin combined with heparin in pregnant women with RPL associated with antiphospholipid antibodies, providing foundational evidence for clinical management strategies.

Another frequently cited study is that of Kutteh (1996), published in the *American Journal of Obstetrics and Gynecology*, which demonstrated the superiority of combined heparin and aspirin therapy over aspirin alone for RPL linked to antiphospholipid antibodies. This study has played a significant role in shaping treatment recommendations.

The RCT conducted by Kaandorp *et al*. (2010), published in the *New England Journal of Medicine*, also had considerable impact, receiving 153 local citations and 328 global citations. This trial further examined the efficacy of aspirin with or without heparin, contributing to evidence-based therapeutic protocols for affected women.

Other notable clinical trials include the following: Farquharson *et al*. (2002) in *Obstetrics and Gynecology* (134 local citations, 356 global citations), which addressed treatment outcomes for antiphospholipid antibody syndrome in pregnancy, and Scottish Pregnancy Intervention study by Clark *et al*. (2010), published in *Blood* (99 local citations, 186 global citations), which evaluated low molecular weight heparin combined with aspirin in RPL patients.

A key systematic review by Empson *et al*. (2005), published in the *Cochrane Database of Systematic Reviews*, synthesized findings from multiple clinical studies and provided a comprehensive evaluation of immunotherapies for RPL in women with antiphospholipid antibodies or lupus anticoagulant (96 local citations, 337 global citations).

In addition to clinical studies, several mechanistic investigations have significantly advanced understanding of disease pathogenesis. The study by Girardi et al. (Girardi *et al.*, 2004) in *Nature Medicine* demonstrated that heparin prevents fetal loss by inhibiting complement activation, offering critical insights into the molecular mechanisms of RPL (87 local citations, 504 global citations).

Other influential studies include the following: Rai *et al*. (1995) in *Human Reproduction* (86 local citations, 207 global citations), which documented high pregnancy loss rates in women with antiphospholipid antibodies; Brenner *et al*. (2000) in *Thrombosis and Hemostasis* (86 local citations, 244 global citations), which evaluated the effectiveness of enoxaparin in preventing RPL; and (Branch *et al*. (1992) in *Obstetrics and Gynecology* (83 local citations, 382 global citations), which assessed outcomes of treated pregnancies in women with antiphospholipid antibody syndrome.

## DISCUSSION

The findings of this bibliometric analysis on the use of immunotherapies in the management of women with RPL indicate a growing and sustained interest in the field, which gained prominence in the 1990s with the publication of the first RCTs. This sustained growth can be attributed to advances in diagnostic methods, therapeutic options, and a greater understanding of the immunological mechanisms underlying pregnancy loss. Approximately two-thirds of the publications identified were original research articles, underscoring the central role of primary studies in expanding the knowledge base of this domain.

The upward trend in the number of publications aligns with the global increase in scientific output in recent decades. As reported in the *UNESCO Science Report*, global scientific production increased by 21% between 2015 and 2019 (UNESCO, 2021). However, this rapid expansion also raises concerns regarding publication quality, as the increasing volume may overwhelm peer review systems and challenge the maintenance of rigorous scientific standards (Zheng *et al.*, 2023).

The leading journals publishing research on immunotherapies for RPL are primarily in the fields of reproductive medicine and reproductive immunology, as well as well-established clinical journals focused on gynecology and obstetrics. A subset of publications also appears in journals specializing in hematology and rheumatology, indicating interdisciplinary connections between RPL and hematologic (e.g., coagulation disorders) and rheumatologic conditions. Notably, the journals with the highest impact factors were those with a focus on assisted reproduction and maternal-fetal medicine.

The analysis of influential authors highlights considerable scientific engagement, though a relatively small group of researchers accounts for a significant proportion of publications. The increasing participation of early-career researchers suggests a dynamic and evolving field with emerging leaders. The United States dominates in terms of scientific production, while the United Kingdom and Canada are distinguished by the high impact of their publications. Germany has a strong profile of international collaboration, while countries such as China, Japan, and Iran exhibit robust research output but with less impact and limited cross-border engagement. This pattern underscores the role of international collaboration in enhancing the visibility and influence of research. Notably, six countries were responsible for 50% of the publications in this field, consistent with trends observed across other scientific disciplines (Nature, 2024).

Keyword analysis revealed evolving research priorities and emerging therapeutic targets. A growing focus on immunological mechanisms—such as the role of NK cells and alterations in humoral and cellular immune responses—reflects increasing sophistication in the understanding of pregnancy complications. The three main immunotherapies consistently identified through keyword mapping were heparin, aspirin, and IVIG. Heparin and aspirin are established treatments, particularly for RPL associated with APS, while IVIG is increasingly used in cases associated with immune abnormalities or unexplained etiology (ESHRE Guideline Group on RPL *et al.*, 2023).

Other therapies, including corticosteroids, lymphocyte immunotherapy, and HCQ, appear to offer potential benefits in improving live birth rates, although the evidence remains limited (de Moreuil *et al.*, 2020; Cavalcante *et al.*, 2023a; Dernoncourt *et al.*, 2024). Emerging treatments such as lipid emulsions and calcineurin inhibitors show promise but were underrepresented in the keyword analysis, likely due to the preliminary nature of current clinical investigations (Cavalcante *et al.*, 2023a; Cavalcante *et al.*, 2023b).

The list of the most influential publications further supports the keyword findings, particularly regarding the widespread use of anticoagulants in APS-related RPL. RCTs provided foundational evidence for the use of heparin and aspirin, while systematic reviews confirmed the robustness of this evidence base. Additionally, mechanistic studies have clarified the underlying pathophysiological processes, facilitating therapeutic advances and improving clinical management strategies.

In conclusion, this bibliometric analysis offers a comprehensive overview of global research trends in the use of immunotherapies for RPL. It highlights a marked increase in research activity and citations, reflecting the growing impact of the field. Key contributors include high impact journals, influential authors, and leading countries such as the United States, China, and the United Kingdom. The evolution of research themes - from early empirical studies to more sophisticated investigations of genetic, immunologic, and molecular mechanisms - demonstrates significant progress. Among the most studied therapies are heparin, aspirin, and IVIG, with novel interventions requiring further validation through well-designed clinical trials. This study emphasizes the importance of international collaboration and the need for continued research to close existing knowledge gaps and enhance therapeutic outcomes for women affected by RPL.
